# Supplementary material for: Identification of photoperiod-induced specific miRNAs in the adrenal glands of Sunite sheep (Ovis aries)
Source: Front Vet Sci. 2022 Jul 22;9:888207. doi: 10.3389/fvets.2022.888207 (PMC9354845; doi:10.3389/fvets.2022.888207)
Supplement: Supplementary file 1 [file Data_Sheet_1.ZIP › Supplementary file/miRDeep2 file.zip!/miRDeep2 file/Know_Structure/SP42A2/oar-mir-30b.pdf]

miRBase precursor	: oar-mir-30b
Total read count	: 38458
oar-miR-30b read count	: 38148
remaining reads	: 310

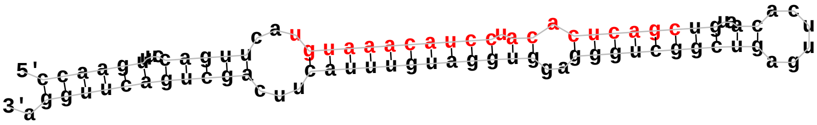

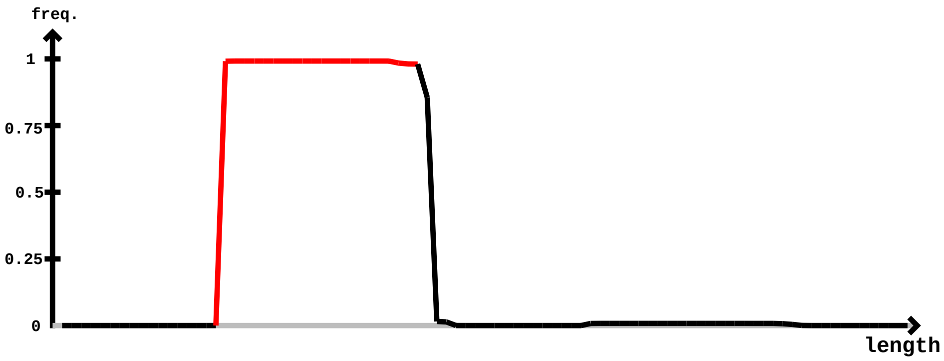


```
                oar-miR-30b
5'- ccaaguuuccaguucauguaaacauccuacacucagcuguaacacuugagucggcugggagguggauguuuacuucagcugacuugga   -3'    exp
    (((((...(((((...((((((((((.((.((((((((..((......))))))))))..))))))))))))...)))))))))))).    reads   mm    sample
    ...............auguaaacauccuacacucagcu..................................................    3       0     seq
    ................uguaaacauccuUcacu.......................................................    1       1     seq
    ................uguaaacauccuacacu.......................................................    4       0     seq
    ................uguaaacauccuacacuU......................................................    64      1     seq
    ................uguaaacaCccuacacuc......................................................    3       1     seq
    ................uguaaacauccuacacuc......................................................    217     0     seq
    ................uguaaacauccuacacuca.....................................................    131     0     seq
    ................uguaaacauccuacacucag....................................................    18      0     seq
    ................uguaaacauccuacacucagc...................................................    4797    0     seq
    ................uguaaacauccuacacucagcu..................................................    32297   0     seq
    ................uguaaacauccuacacucagcug.................................................    69      0     seq
    ................uguaaacauccuacacucagcugu................................................    515     0     seq
    ................uguaaacauccuacacucagcugua...............................................    3       0     seq
    .................guaaacauccuacacuc......................................................    2       0     seq
    .................guaaacauccuacacucagc...................................................    3       0     seq
    .................guaaacauccuacacucagcu..................................................    17      0     seq
    .................guaaacauccuacacucagcugu................................................    3       0     seq
    ..................uaaacauccuacacucagcugu................................................    1       0     seq
    ......................................................cugggagguggauguuu.................    1       0     seq
    ......................................................cugggagguggauguuuac...............    2       0     seq
    ......................................................cugggagguggauguuuacu..............    41      0     seq
    ......................................................cugggagguggauguuuacuu.............    94      0     seq
    ......................................................cugggagguggauguuuacuuc............    149     0     seq
    ......................................................cugggagguggauguuuacuuca...........    14      0     seq
    .......................................................ugggagguggauguuuacu..............    1       0     seq
    .......................................................ugggagguggauguuuacuu.............    3       0     seq
    .......................................................ugggagguggauguuuacuuc............    4       0     seq
    .......................................................ugggagguggauguuuacuuca...........    1       0     seq
```
